# Why motor imagery is not really motoric: towards a re-conceptualization in terms of effect-based action control

**DOI:** 10.1007/s00426-022-01773-w

**Published:** 2022-12-14

**Authors:** Patric Bach, Cornelia Frank, Wilfried Kunde

**Affiliations:** 1 School of Psychology, University of Aberdeen, William Guild Building, Kings College, Aberdeen, UK; 2 Department of Sports and Movement Science, School of Educational and Cultural Studies, Osnabrück University, Osnabrück, Germany; 3 Department of Psychology, Julius-Maximilians-Universität Würzburg, Röntgenring 11, Würzburg, Germany

## Abstract

Overt and imagined action seem inextricably linked. Both have similar timing, activate shared brain circuits, and motor imagery influences overt action and vice versa. Motor imagery is, therefore, often assumed to recruit the same motor processes that govern action execution, and which allow one to play through or simulate actions offline. Here, we advance a very different conceptualization. Accordingly, the links between imagery and overt action do not arise because action imagery is intrinsically motoric, but because action planning is intrinsically imaginistic and occurs in terms of the perceptual effects one want to achieve. Seen like this, the term ‘motor imagery’ is a misnomer of what is more appropriately portrayed as ‘effect imagery’. In this article, we review the long-standing arguments for effect-based accounts of action, which are often ignored in motor imagery research. We show that such views provide a straightforward account of motor imagery. We review the evidence for imagery-execution overlaps through this new lens and argue that they indeed emerge because every action we execute is planned, initiated and controlled through an imagery-like process. We highlight findings that this new view can now explain and point out open questions.

## Introduction

Overt and imagined action seems inextricably linked. Before undertaking a difficult motor task, people often experience themselves imagining what they intend to do, and the form this imagination takes (e.g., imagining intended outcomes or motor behaviors) affects task success and subsequent learning (e.g., Land et al., 2014; Woolfolk et al., 1985a, 1985b). Sometimes, people even imagine behaviors they will execute at a much later time and in a different environment, for example, when they mentally play through the actions of their sport from the privacy of their home. Again, this form of motor imagery—sometimes termed mental practice, mental training or motor imagery training (Schack et al., 2014; for definitions and conceptualizations, see Morris et al., 2005)—affects later performance (for meta-analysis, Driskell et al., 1994; Simonsmeier et al., 2021; Toth et al., 2020) and is recommended by most professional coaches (Mayer & Hermann, 2019). Purely mental practice can even increase measured muscle strength, from simple finger contractions to leg pressing and triceps extension, albeit not to the same extent as physical practice (Yue & Cole, 1992; for recent replications and review, see Paravlik et al., 2018; Reiser et al., 2011; Smith et al., 2003).

Studies from experimental psychology and cognitive neuroscience support this coupling of overt and imagined action. There are tight correspondences between the timing of imagined and overt actions (Decety et al., 1989; Wohlschläger & Wohlschläger, 1998; for a critical review, see Guillot & Collet, 2005), between the activated brain structures in parietal and premotor cortices (for reviews, see Lotze & Halsband, 2006; Hétu et al., 2013; O’Shea & Moran, 2017), and between the lawful regularities that govern the kinematics of both overt and imagined action (e.g., Fitts’ law, Decety & Jeannerod, 1995; two-thirds power law, Karklinsky & Flash, 2015; Papaxanthis et al., 2012). Moreover, several studies show that motor imagery can engender (sub-threshold) activation in the muscles used in the imagined behavior (Guillot et al., 2007, 2010; Jacobson, 1931, 1932; Lutz, 2003; Munzert & Krüger, 2018; Shaw, 1938), and, conversely, that executing motor actions makes imagining the same actions easier and imagining different actions harder (e.g., Wohlschläger, 1996, 2001; Callow et al., 2006; Guillot et al., 2013; for a broader review of the effects of such “dynamic motor imagery”, see Guillot, under review). The link from imagined to overt behavior is so strong that it provides the basis for several (stage) magical phenomena. In Chevreul’s pendulum and the Ouija board, for example, seemingly supernatural motions happen simply because participants’ imagined motions are, unbeknownst to them, translated into subliminal hand and finger movements that are made visible by the devices (Cantergi et al., 2021; Chevreul, 1833; Easton & Shor, 1975, 1976, 1977; Wegner et al., 1998).

A standard explanation for these findings is that imagery of action is an intrinsically motoric process. This view assumes that motor imagery, in a form of *neural re-use* (e.g., Anderson, 2010), draws upon the same neuronal networks and cognitive processes that underlie action execution itself (Jeannerod, 1994; Jeannerod & Decety, 1995). As a potential mechanism, it has been proposed that the brain predicts—via forward models—the sensory consequences that each of its motor commands will produce, so that it can anticipate the visual, tactile, and proprioceptive sensations that will soon be registered (e.g., Miall & Wolpert, 1996; Sperry, 1950). During overt action, such predictions may allow the actor to filter out predicted sensations (e.g., Reichenbach et al., 2014) or to correct for movement errors before they happen (e.g., Desmurget & Grafton, 2000; Shadmehr et al., 2010). During imagery, the same forward models could be used offline, triggered perhaps by sub-threshold motor commands, and allow one to mentally play through how different actions will unfold, without the signals ever reaching the muscles (e.g., Jeannerod, 1994; Jeannerod & Decety, 1995; Kilteni et al., 2018).

In these proposals, motor imagery is often described as “neural simulation of action” (e.g., Jeannerod, 2001), “covert execution” (e.g., Scheil et al., 2020), and imagined actions are taken as “real actions, except for the fact that they are not executed” (Jeannerod, 2001, p. 103). In essence, these accounts hold that people can imagine their actions because the motoric structures of the brain can, in some form, pretend that the imagined actions are currently executed, and project their perceptual consequences into the imagination, so that one can watch them unfold in front of one’s mind’s eye. Imagination, therefore, has the same timing, is governed by the same regularities, and activates largely overlapping brain structures as overt action.

## A different view on motor imagery

The previous section describes the “standard” conception of motor imagery. In this article, we would like to advance a markedly different conceptualization, which turns the proposed relationship on its head, and which—as we argue further below—provides a closer match to the extant data. On this view, the links between imagery and overt action do not arise because action imagery is intrinsically motoric, but—conversely—because the mechanisms people use to control their voluntary behavior are intrinsically imaginistic. In other words, the observed overlaps emerge not because motor imagery recruits motor-based resources but because every action we execute is planned, initiated and controlled through imagery (e.g., Colton et al., 2018; Hommel, 2009; Hommel et al., 2001; Janczyk & Kunde, 2020; Pfister, 2019; Pfister, 2019; Pfister et al., 2014; Prinz, 1997; Shin et al., 2010).

This proposal is not new. In fact, it is the classic solution of the ideomotor theorists (e.g., Carpenter, 1852; Harleß, 1861; James, 1890; Lotze, 1852) to the puzzle of how people can achieve voluntary control over their body movements, given that they have very little actual insight into the actual working of their motor apparatus. Even the simplest act of reaching and grasping requires complex coordination of a multitude of muscles, most of which a person is not aware of, including those in one’s back that prevent one from falling forward when extending one’s arm. The reader could ask themselves, for example, where in fact the muscles they use to control their finger movements are located, what produces the sounds when they snap their fingers, or how they make a bicycle go left or right. Surprisingly, most people answer these questions incorrectly, even though they have performed the actions many times (see^1^ for answers). This “executive ignorance” (Turvey, 1977) into the motor activities that make up our daily lives was neatly summarized by William James: “we are only conversant with the outward results of our volition, and not with the hidden inner machinery of nerves and muscles which are what primarily sets it at work” (1890, p. 499).

Ideomotor theorists argue that imagery is the trick that people use to gain control of a motor apparatus that is essentially a black box to them. The idea is that people do not try to—and actually cannot—directly control their “hidden machinery of nerves of muscles” (James, 1890, p. 499). They can only ever bring to mind a mental image of the “outward results” that they want to achieve, which then activates the motor patterns that will bring this result about. The mechanisms that are assumed to underlie this transformation of imagery into action are surprisingly simple and can be accounted for by established laws of associative learning. Accordingly, human agents learn to link, through a lifetime process of self-observation, the different efferent/motor activities that they produce, at first accidentally (‘motor babbling’), with the perceptual effects these activities reliably cause (Hommel & Elsner, 2001; James, 1890; Prinz, 1997). In short, they learn how their body movements look, feel, and sound, and how they affect the environment. The main argument is that as soon as a behavior and its likely effects are robustly associated, the mere *intention* to produce any perceivable effect activates the motor pattern to which it was associated by previous experience. Thus people can purposefully control their black box motor apparatus, merely by thinking of the perceptual effects they wish to achieve: we imagine what it looks like to move our fingers, we bring to mind the sound of our fingers snapping, or where we want our bicycle to go next—and by the previously formed associations the corresponding efferent activities are recollected, without us ever needing to know how they internally realized: imagery of the intended “outward results” is enough to elicit the motor behaviors itself.

While such proposals of effect-based action control have been only rarely connected to the phenomenon of motor imagery, they form the core of modern accounts of effect-based action control (for reviews, see Shin et al., 2010; Pfister, 2019), and are captured by recent ideas of action control through internal (inverse and forward) models (Wolpert, 1997) and predictive coding/active inference (e.g., Adams et al., 2013). They are supported by a large body of evidence. A full review is not possible here (see Pfister, 2019), but studies have shown that any manipulation that helps people bring to mind the effects of their actions indeed activates the motor behaviors themselves, in line with the idea that both are closely associated. For example, participants execute actions more quickly when primed, just before execution, with an image of the finger (or other body part) movements they should make (e.g., Bach et al., 2007; Brass et al., 2001), even if this prime is only anticipated but not perceived (Kunde et al., 2004; see Badets et al., 2016 for review). In some cases, this priming is enough to inadvertently cause participants to execute actions they have been asked to withhold (Colton et al., 2018). Even distal perceptual consequences of one’s actions (like a sound elicited by a button press) prime the motor behaviors that usually bring them about, provided they have been robustly associated before (e.g., Hommel & Elsner, 2001; Ziessler et al., 2012).

Neuroimaging research is also consistent with the proposed tight coupling of motor and effect-related representations. In the last decades, it has become clear that, throughout the cortical hierarchy, neuronal populations often code for both, efferent (motor) activities and the afferent (perceptual) states they produce, to the extent that it has become increasingly difficult to delineate purely afferent or efferent regions. Regions in early and late visual cortex, for example, previously thought to play purely perceptual roles, have been found to be involved in action planning, and specifically to encode the effects one wants to cause with one’s actions (Kühn et al., 2011; van Steenbergen et al., 2017; Zimmermann et al., 2016, 2018). A similar “common coding” (Prinz, 1997) of perceptual and motor components also exists in the parietal lobe (e.g., Monaco et al., 2020; Oosterhof et al., 2012) and the premotor cortex, where neurons have been identified that code actions equally when they are executed and when their effects are perceived, both visually and auditorily (i.e., “mirror neurons”, di Pellegrino et al., 1992; Gallese et al., 1996; Kohler, et al., 2002). Even the primary motor cortex, often implicated in motor output exclusively, seems to play perceptual roles by representing the anticipated proprioceptive/kinesthetic consequences of one’s behavior (de Lange et al., 2013; Gandolla et al., 2014; Naito, 2004). Indeed, it has been argued that the motor commands it sends to the spinal cord may be, in fact, nothing else than proprioceptively coded goal states for one’s limbs (e.g., Adams et al., 2013).

Together, therefore, there is ample evidence from both experimental psychology and neuroscience that actions are represented in terms of their intended effects, which then activate the motor behaviors to which they are associated. Recent proposals have extended this simple associative architecture towards alternative mechanisms for acquiring motor-perceptual links (e.g., propositional relationships, Sun et al., 2022), describe how they can account for complex, hierarchical or multi-step actions (e.g., Kachergis et al., 2014; Moeller & Frings, 2019; Moeller & Pfister, 2022), and how motor behavior can be dynamically adjusted in response to error, when actual motor output diverges from the intended goal states (Adams et al., 2013; Kunde et al., 2017; Wolpert, 1997).

If these ideas of effect-based action control are taken seriously, they lead to a subtle but—in our minds—illuminating re-conceptualization of motor imagery. Motor imagery, in such frameworks, does not rely on neural re-use of execution-related (efferent) structures in the brain, but instead reflects specifically the perceptual process through which people plan, initiate and control their action: the bringing to mind of the goal states—the effects—an actor wants to achieve: how they want their actions to look, sound and feel, when they are carried out. The difference to overt action is only that, during motor imagery, this imaginistic process is decoupled from the motor apparatus, to prevent the imagined action effects from inadvertently causing overt behavior.

Several—not mutually exclusive—alternatives exist how this decoupling may work. One possibility is that efferent activities are somehow inhibited (e.g.,Berthoz, 1996; Di Rienzo et al., 2014; Guillot et al., 2012; Rieger et al., 2017). Another possibility is that the activation threshold to trigger associated motor patterns is deliberately upregulated to prevent an automatic outflow of efferent activity (see also, Berthoz, 1996). Both alternatives would make it possible for people to plan/imagine actions freely, without being in danger of inadvertently releasing the associated motor behaviors. In fact, this increase of the execution threshold might be the very reason for why agents can become aware of their action imagery at all. During most everyday activities, people move their body without experiencing the mental images they use to control these movements, suggesting that even weak, subliminal action images can drive motor behavior effectively (for evidence, see Kunde, 2004; Linser & Goschke, 2007). From an ideomotor perspective, the upregulation of the motor threshold may therefore be precisely what makes motor imagery possible. It allows people to imagine their actions strongly enough to be consciously experienced while still not eliciting overt behavior.

A recent series of experiments (Colton et al., 2018) tested the idea that imagined actions are planned actions that are activated just below the execution threshold. Participants were asked to imagine—but not execute—different sequences of finger movements. In some trials, we unexpectedly strengthened this imagination through a visual cue that showed the effects of the action they currently imagined (e.g., a specific finger depressing), in the hope that this surprising additional activation would drive the action super-threshold and cause its involuntary release. This is exactly what was found. When imagery and visual cues were congruent and could therefore combine, participants found themselves sometimes executing actions they were asked to withhold. If, however, the visual cue did not match what was imagined, the likelihood of an accidental action slip was reduced compared to baseline (for similar evidence, see Kunde et al., 2004; Maslovat et al., 2013).

Other studies show that overt action can be effectively influenced even when the relevant action images are weak and remain subliminal, for example, when very briefly presented before participants make a relevant motor response (e.g., Kunde, 2004; Linser & Goschke, 2007). Importantly, a recent study showed that vividly imagined actions have stronger effects on subsequent motor behavior than even actions that have been explicitly prepared for action (Toovey et al., 2021). This is consistent with the idea that motor imagery involves a hyper-activation of the movement images that people use to control their behavior so that they can be consciously experienced, while at the same time being prevented from causing motor output.

This conceptualization of motor imagery as (hyper-activated) effect imagery differs from conventional accounts in that it does not require information from output-related components of the motor apparatus. Instead, it conceptualizes motor imagery simply as the planning-related processes that otherwise shape’s people overt motor behavior (see also Glover & Baran, 2017; Jeannerod, 1994; Toovey et al., 2021) by specifying its goals as well as those more proximal components that are important for action success (e.g., movements speeds, specific trajectories around goals, etc.)^2^. In other words, human agents can imagine actions because they have done them over and over in their daily life and have internalized how they look, sound, and feel. From then on, they can then simply recall this perceptual knowledge to produce a vivid experience of the actions they want to imagine.

Importantly, while this account does not require a contribution of motoric knowledge, it does not imply that imagery cannot make use of it. In a radical ideomotor view, efferent (“motor”) activities are bidirectionally associated to the effects they will cause (cf. Hecht et al., 2001; Hommel and Elsner, 2001; Hommel et al., 2001). Via these links, imagining or intending a specific effect can bring about the associated motor behaviors, but—once they are selected—the activated motor behaviors can also activate the effects they will most likely cause. This is functionally important because the effects used to select a motor behavior and those it will cause do not need to be identical; any action will bring with itself changes that have not been explicitly intended. For example, one may intend to grasp a coffee mug, by anticipating the required visual and proprioceptive components of the movement trajectory towards the goal, as well as the specific points of contact with the mug, but without anticipating the tactile experience of one’s pullover’s sleeve moving across the forearm, when carrying out that movement.

Through these links, any motor behavior that is activated may, therefore, enrich or further constrain imagery with what is most likely to happen when it is executed. This associative activation of the effects an action will *also* cause might be called a “prediction” and is functionally equivalent to the output of a forward model (cf. Kawato, 1999). Such a bi-directional associative architecture, therefore, fully implements the main functional mechanism of conventional accounts of imagery, through which (subliminal) motor activation can project ahead which perceptual effects it will most likely cause. The difference is that, in effect-based accounts, this motor-based prediction is not the default mode. Instead, it is considered as an additional, optional step, that one can draw upon only after motor activities have been accessed through the intended perceptual changes in the first place. There is still no pure “motoric” imagination that is not triggered by a prior imagination of the intended action outcomes. However, once a specific motor behavior that implements these desired effects is activated, it can be supplemented or adjusted through associations to the effects this motor behavior will also have, if carried out.

We believe that such effect-based accounts of motor imagery solve (or sidestep) most problems and inconsistencies in standard accounts. Note for example that, in standard accounts, imagery emerges from motor commands being fed to a forward model-like mechanism, which then plays through the sequence of perceptual consequences these actions will achieve. What is usually left open is where these motor commands come from in the first place. Why, if one already knows the sequence of movements one wants to imagine, is it necessary (a) to identify the precise motor command that brings about this imagination, and (b) how is the relevant chain of motor commands selected, so that it matches this (sometimes quite complex) imagination goal? This ambiguity is even more pronounced if one considers that the mechanisms (i.e., forward models, efference copies) that are proposed to serve as the basis for this imagination are usually assumed to have evolved to predict the outcomes of actions that the organism does indeed execute (i.e., for anticipatory error control or filtering out expected stimulation). How then is it possible (c) to fool these mechanisms into completing the same job for actions that one does decidedly does not want to execute, because one only wants to imagine them? Effect-based accounts of motor imagery solve, or sidestep, all these problems by providing a straightforward associative account of how action imagery can affect the motor apparatus, and how, in turn, motoric activity can feed back into what people imagine.

## Accounting for the evidence

We believe that a re-formulation of motor imagery as action planning decoupled from execution has various conceptual advantages over standard accounts. Here, we briefly review how the major findings, which are usually taken as evidence for the standard account, are explained by effect-based accounts of motor imagery.

### Motor imagery elicits muscular activity

One central finding is that motor imagery often engenders measurable activation in the muscles involved in the action (e.g., Guillot et al., 2007; Jacobson, 1931, 1932). The traditional explanation is that this shows the working of an execution-related process, for example, the subliminal activation of motor routines that—via forward modeling—supply imagery with the relevant “material” to play through, such as the precise kinematics or timing of the action one imagines. In effect-based accounts, the explanation of this motor outflow is more straightforward. It simply reflects that the neuronal codes that make up one’s imagery of an action are the same ones that one would use—via the previously formed associations—to initiate this action. In such views, it is not surprising that some subliminal activation would persist and reach the relevant muscles, even if the motor threshold itself is not passed and no full action is ever released. In the words of William James, “every mental representation of a movement awakens to some degree the actual movement which is its object” (James, 1890). Thus, while these motor activations may indicate that some form of action imagery takes place, they do not necessarily play a functional role in the imagery process.

Several findings suggest that this is exactly what is going on. Several studies show striking dissociations between imagery and motor processes. It has long been observed, for example, that muscular responses during mental practice are often only loosely related to the actions one tries to imagine (Heuer, 1985), and that motor imagery can elicit motor-like brain activity even if motor output is fully controlled (Zabicki et al., 2017). Moreover, motor imagery has been found to typically capture the (ideal) actions one would *want* to carry out but less so the *actual* motor behavior that results, such as the errors one makes (e.g., Dahm & Rieger, 2019; Rieger et al., 2011a, b) or the variability of one’s movements (Niziolek et al., 2013). Such dissociations are problematic for accounts in which motor processes are the causal driver of action imagery, but they are of course fully in line with effect-based views where imagery is primarily perceptually driven, reflecting one’s prior knowledge of how these behaviors *should* look, sound and feel, and not how they actually do when executed. 

Other studies show that imagery of non-motor behavior is often just as effective in causing overt behavior as that of the motor behavior itself, if this non-motor behavior represents possible effects one can cause. One only needs to take a pendulum in one’s hand and image it is swinging: one’s hand will produce these swings, even though one does not imagine the hand itself moving, and is in fact often unaware that it does so (Cantergi et al., 2021; Chevreul, 1833; Easton & Shor, 1975, 1976, 1977; Wegner et al., 1998). Such findings can be replicated in lab-situations, where visualizing certain shapes is enough to induce a tendency to reproduce these shapes with one’s hands, even if these shapes belong to objects that one never has interacted with before (e.g., the circle shape of the moon), so that any action imagery is unlikely (Bach et al., 2010a). Both findings are much more in line with the idea that motor output during imagery is ultimately driven by bringing to mind the intended action effects, rather than the motor behaviors themselves.

A final example is tool use. For many tools, the effects they produce are clearly dissociable from the motor behaviors that cause them. For example, the Fulcrum effect (Kunde et al., 2007) denotes the fact that in laparoscopic surgery, movements of the hand upwards produce downwards movements of the endoscopic tool, and vice versa (for a review of similar tool transformation findings, see Heuer & Sülzenbrück, 2013). Importantly, when people plan such actions, then bringing to mind the intended motions of the tool suffices to elicit the relevant motor behaviors, even if they are opposite to intended tool motion (Müsseler et al., 2008). For example, imaging a clockwise rotation of an object facilitates counter-clockwise manual rotations of a steering wheel, provided this produces an intended clockwise rotation of the task-relevant visual object (which applies to current displays in aviation, Janczyk et al., 2012). These findings, therefore, provide clear evidence that motor imagery—like action planning—occurs not in terms of motoric outflow, but in terms of the effects one wants to achieve with one’s actions (Hommel et al., 2001; Prinz, 1997), and there is evidence that also the neuronal coding of these actions seems to occur to a large extent in such an effect-based rather than motoric format (e.g., Ferrari et al., 2005).

### Action execution can help or hinder imagery

A second class of important findings is that executing motor behaviors disrupts imagery of different actions, but helps imagery of similar ones (e.g., Wohlschläger, 1996, 2001; Callow et al., 2006; for a review, see Guillot, under review). In the standard view, these effects emerge because carrying out an action occupies one’s execution-related resources so that they cannot be deployed for motor imagery, or because a forward model that is fed by mismatching motor commands will also conjure up mismatching mental images. In principle, these explanations can also work in frameworks of effect-based action control, as they assume not only that effect imagery triggers motor behavior, but also that motor behavior, in turn, is associated to the relevant perceptual effects it will bring about (e.g., Hommel et al., 2001; Müsseler & Hommel, 1997). Executing movements that differ from imagination will therefore by necessity also conjure up inappropriate effect images and interfere with the imagery process.

However, we suspect that there might be a simpler explanation for many of these findings. Executing a movement is not perception-free. Even if people are not looking at the body parts they move, they still receive (proprioceptive, tactile, etc.) feedback from their limbs and muscles. And of course, this feedback can also be congruent or incongruent with the perceptual effects they imagine, so that any interference can arise not only from predicted feedback, but also from the actual feedback that is inconsistent imagination. A classic example is that people can mentally rotate an object more easily if they rotate their hands in the same direction, compared to the opposite direction (Wohlschläger, 1996, 2001). This does not necessarily reflect the anticipation of incongruent movement effects, but also the multisensory associations of clockwise proprioceptive feedback with clockwise visual motions (cf. de Lange et al., 2006; Shenton et al., 2004).

Findings from deafferented patients—who receive no proprioceptive feedback—provide direct evidence for such multisensory associations. Usually, people find it difficult to draw when they only see the mirror image of their drawing hand, as there is a mismatch between visual and proprioceptive information: leftward hand movements look rightwards in the mirror and vice versa. To compensate, neurotypical agents often try to ignore such mismatching proprioceptive feedback, causing ‘functional haptic neglect’ (Heuer & Rapp, 2012; Liesner & Kunde, 2020)*.* Strikingly, however, the problems with mirror drawing are absent in deafferented patients, showing that the interference indeed largely emerges from a perceptual (proprioceptive to visual) locus (Lajoie et al., 1992).

### Temporal and kinematic similarities between imagery and action

A central piece of evidence for motoric accounts is the tight temporal link between overt and imagined action (e.g., Decety et al., 1989; Wohlschläger & Wohlschläger, 1998) and the finding that both are governed by similar regularities, such as Fitts’ law, or the two-thirds Power law between speed and curvature of biological movements (Decety & Jeannerod, 1995; Karklinsky & Flash, 2015; Papaxanthis et al, 2012). As these constraints are argued to emerge from the motor system, their re-occurrence in imagery is taken as evidence that imagery must reflect a “read out” of a motoric process.

Please note that such findings can be accommodated in effect-based accounts, if one assumes that not only activated action images can activate the efferent activities that bring them about (i.e., the inverse route), but also that activated motor actions can activate the perceptual effects they produce (i.e., the forward route), and in doing so can retrieve perceptual consequences that are not fully specified in the original action plan (see above). Nevertheless, we do believe that many of the findings that are usually provided as evidence for such influences should not be taken at face value. Typical research participants have ample perceptual experience with their own behavior (and that of others) throughout their lifetime, and it would be no surprise that their imagination—and ability to plan their actions—has internalized these regularities and takes them into account. A strong case for motoric readout could therefore only be made in cases where imagery can be shown to be governed by a regularity that (a) unambiguously reflects constraints from the motor apparatus, and which (b) participants had no prior perceptual experience with. We are not aware of any such demonstration (but see Rieger, Boe, Ingram, Bart, Dahm, under review, this volume, for a different view**)**. Instead, several studies have suggested that regularities originally attributed to the motor apparatus may in fact reflect perceptual or imaginistic constraints. For example, it is well-established that people find it almost impossible to perform particular irregular bimanual movements, for example when having to describe three circles with one hand while the other hand performs four (Kelso, 1995). These limitations have been put down to constraints of the motor apparatus, such as a synchronization of motor commands in the spinal cord. However, in an impressive series of demonstrations, Mechsner and colleagues (Mechsner, 2003; Mechsner et al., 2001) have shown that these phenomena may instead reflect constraints of imaginistic action planning. The limitations were alleviated when participants were given help to perceptually coordinate—imagine—these actions, so that “impossible” movements suddenly became possible (for an earlier demonstration of similar effects, see Swinnen et al., 1997; for a review of related effects, see Shea et al., 2016).

In addition, many of the initial findings of tight temporal overlaps between overt and imagined actions have been recently challenged, with several showing either consistent over- or under-estimation of the timings of imagined actions, for different task and stimulus contexts (e.g., Reed, 2002; for a review, see Guillot & Collet, 2005). This temporal elasticity is problematic for approaches that assume that people’s mental motor imagery emerges from a subliminal engagement of the underlying motor routines, which should then closely match their actual time course. However, if imagery reflects a planning process that is decoupled from execution (see also Jeannerod, 1994; Glover & Baran, 2017), such differences are not surprising, and indeed expected, if one assumes that people flexibly decide to either faithfully follow their memories of action execution, or go through their major functional stages only, or even speed up or slow down specific steps to extract the information required for their particular imagery goal.

### Imagery and action activate similar brain regions

A final important piece of evidence for motoric accounts is the tight overlap between brain structures activated by overt and imagined action (for reviews, see Lotze & Halsband, 2006; O’Shea & Moran, 2017). In our view, an interesting, but often ignored, aspect of these observations is that this overlap is primarily observed in structures associated with action planning—like premotor and parietal cortices—but it is much less robust in execution-related structures, like the primary motor cortices, basal ganglia and cerebellum (for review and meta-analysis, see O’Shea & Moran, 2017; Hétu et al., 2013; Munzert et al., 2009). For motoric accounts of action imagery, such a finding should be puzzling. Yet, it is exactly what would be predicted by accounts of effect-based action control, where action imagery is conceptualized precisely as a shift towards planning away from execution-related resources (see also Jeannerod, 1994; Baran & Glover, 2017).

An elegant study confirmed this link between motor imagery and planning-related instead of execution-related processes, while controlling for actual body movement (Raffin et al., 2012). The authors asked amputees, who still subjectively felt the presence of their phantom limbs, to either really move their absent limbs or imagine moving them. No movement—and no proprioceptive feedback—was of course present in either case. Nevertheless, there were clear differences in brain activation. The execution instruction more strongly activated primary somatosensory and motor cortices and the anterior lobe of the cerebellum. In contrast, during imagination, there was more activity in the parietal and occipital lobes, and the posterior cerebellum (see also Hanakawa et al., 2008). Importantly, it was found that these regions matched those when the patients really moved, or only imagined moving, their still intact limbs on the other side. This shows both: that motor execution and imagery are indeed different, and that these differences map onto the proposed shift from motor to planning-related resources of effect-based control.

## Open questions and outlook

Motoric views of motor imagery have left several puzzles open that have either been unresolved, or not been addressed. An effect-based view provides a new view on them, and—in many cases—clear avenues how they can be resolved, including testable predictions. Below, we provide a first sketch for some of these open questions.

### When does imagery draw upon motor resources?

In effect-based accounts, motor imagery primarily reflects the planning stages of actions, that is, the bringing to mind of how the target actions would manifest perceptually, based on one’s prior experience with them. In contrast to conventional accounts, there is usually no need for imagery to draw upon knowledge encoded by the motor apparatus, for example, by accessing a forward model that is fed with the motor commands that would generate the behaviors one wants to imagine. As pointed out above, this does not mean that such an influence is not possible, however. The simple associative architecture of effect-based control predicts not only that the effects one intends will elicit the motor behaviors that will bring these effects about, but also that—via the same associations—the motor behaviors, once selected, activate the effects they will produce (e.g., Hommel et al., 2001; Müsseler & Hommel, 1997). These latter pathways, therefore, allow one to derive how one’s behaviors will play out, providing a secondary “motoric” path to imagery.

An important question is under what circumstances this secondary route is made use of. We suspect that this happens specifically when the effects usually used to plan a particular action do not correspond to the information required by the current imagery task. For example, it is typically assumed that the effects agents use to control their behavior are coded in a relatively sparse manner, capturing mainly the intended “distal” bodily or environmental effects (Hommel et al., 2001), the required transitions between current and intended perceptual states (Kunde et al., 2017), or the major functional sub-steps of an action sequence (Schack & Mechsner, 2006). As the specific timing and kinematic of one’s skilled movements emerge, to a large extent, from biomechanical constraints within the muscular and skeletal system during execution, they are most likely not represented on this planning level, unless the skill specifically requires controlling them (e.g., in dance, or for unusual actions cf., Mechsner et al., 2001). If this is correct, then the motoric route might be necessary whenever the imagery task requires access to action features that are not captured by the perceptual goal states people use to control their actions.

Accordingly, for motor behavior where timing and kinematics do not need accurate endogenous controlled, imagery should similarly only engender limited activation in brain structures involved in action execution, and outflow to muscles should be limited. However, as soon as one’s imagery task requires deriving accurate timing, precise kinematics, or access to internal bodily feedback, such motoric effects should emerge also for these movements. Several studies show that motoric indices of motor imagery indeed emerge for such instructions (e.g., Stinear et al., 2006), and this notion is also well supported by the implicit practice of motor imagery researchers. To ensure that motoric activations are found, researchers often instruct their participants to focus on the proprioception and kinesthesis, or the sensations of the actions, not just their visual representation, or tasks are made sufficiently complex to require such forward modeling.

### Why is mental practice effective?

One rarely addressed conflict in standard approaches is the evidence that mental practice is particularly effective in conditions in which it should not be. If motor imagery practice relies on a subliminal playthrough of motor routines, why is it more effective in sports with strong cognitive instead of motor components (such as chess and golf; see Ryan & Simons, 1981, 1983 for the original distinction), why are effect sizes usually smaller for purely motoric ones (for meta-analyses, see Driskell et al., 1994; Toth et al., 2020; Weinberg, 2008), and why is the myoelectric activity that accompanies it only loosely connected to the practiced movements (Heuer, 1985)? The findings are similar for studies that test how learners are best instructed in a motor skill. It is usually better to instruct people in terms of the consequences—the effects—they want to achieve their actions (e.g., focus on hitting the darts board in the bullseye) than reminding them of the proper motoric technique (pulling back the hand, flick of the finger, etc.), which can even be counter-productive (for review, see Wulf, 2013).

If one assumes that mental practice is effective because it affords a subliminal playthrough of motor routines, stronger benefits in the absence of motoric instructions should be at least slightly puzzling. However, such results are of course precisely what would be expected under and effects-based imagery view. In such views, playing through one’s action would simply help in deriving better plans for one’s actions, without necessary input from efferent/motor activities. Repeated effect imagery alone can (a) make these effect images more accessible so that they are more easily brought to mind in the performance situation, it (b) can make these images sharper and more salient so that they become better drivers of muscular activities, and (c), especially for longer action sequences, it might help one to imagine different sub-steps and schedule them more efficiently.

Consistent with these views, recent work shows that athletes represent skilled actions (such as a golf putt) in a hierarchical manner (Schack & Mechsner, 2006). These perceptual-cognitive representations describe the interrelations between the action’s functional parts, each of them being linked to particular perceptual effects that need to be achieved by that component of the motor skill (Bläsing et al., 2009; Schack, 2004, 2020). When novices physically practice these skills, these representations become stabilized and structurally revised into functional groups (e.g., a preparation phase, a clubhead-ball impact phase, and an attenuation phase after the putt; Frank, 2016; Frank et al., 2013).

From an effect-based view, mental practice can similarly stabilize these acquired skill structures and cause similar revisions as physical practice (Frank, 2014; Schack, 2006; Schack & Frank, 2019). Indeed, when novices practice mentally, their perceptual-cognitive representation system shows changes to a skill’s cognitive representation that match those after physical practice (Frank et al., 2014). Strikingly, however, while changes in novices’ representations also lead to overt behavioral changes after physical practice, they do not do so necessarily after mental practice; they only emerge if athletes had some prior physical experience in which the different parts of a skill’s structure were associated with the motor activities that produce these effects (Frank et al., 2014). In this way, the effect-based approach suggests some clear boundary conditions when mental practice should be effective, pointing specifically to the requirement of pre-existing ideomotor associations that can link the mentally practiced effects to the efferent activities that would achieve them (i.e., an inverse model).

An interesting avenue for future research is attempts to enrich actions by additional perceptual feedback (e.g., sonification for the case of auditory feedback, e.g., Effenberg et al., 2016). The idea here is to provide agents with auditory feedback of their body movements that is more discriminable than the perceptual (visual, interoceptive) feedback these movements naturally produce. This should not only enable agents to distinguish motor patterns that were not discriminable without such feedback, but also provide them with another means to imagine their movements. They can imagine how it sounds to move in specific ways, rather than just visually or proprioceptively imagine doing so, just as musicians plan their playing in terms of these musical effects instead of body movements (Brooks, 1995; Drost et al., 2005a, 2005b).

### Integration of motor with non-motor imagery?

Most everyday tasks require the integration of own action capabilities with the characteristics of the environment. For example, for a successful golf swing, the golfer needs to coordinate their (upper) body and limbs with respect to the grass surface, the club they use, direction and speed of the wind, among other factors. Similarly, during everyday tool use, people need to consider motoric features (e.g., the flexion and extension of arms during hammering), relative to the mechanical properties of the tool and the object it is applied to (e.g., a nail vs. a pane of glass) (see Osiurak & Badets, 2016, for a review of mechanical problem solving in tool use).

An account that can describe how such actions are planned, or imagined, needs a means of integrating motoric with extra-motoric information form the physical and social environment (see for similar ideas in an action observation context). It is difficult to see how such integration would be implemented in conventional accounts of motor imagery that rely on a motor-to-perceptual route only, as it is unclear how non-motoric knowledge about the physical behavior of objects could interact with motoric knowledge of one’s own action (but see Schubotz, 2007 for an interesting approach). However, if one assumes, as effect-based accounts do, that action planning generally relies on the same cognitive codes as imagery of physical events, such an integration is no mystery. One’s perceptual learning about the behavior of physical system and about one’s own motor behavior can be seamlessly integrated (see Prinz, 1992, for a similar argument in action control).

Some recent evidence from monkey single cell recordings studies shows that the perceptual systems indeed support such integration. In one study, monkeys first had to identify a relevant object, then trace its outline, and then direct an action towards its endpoint. Early visual cortex played a central role in each of these steps, initially highlighting the relevant object, then amplifying its outline, and then its endpoint, which then served as the basis for eye movements towards it (Moro et al., 2010, Roelfsema et al., 2003; for a review, see Roelfsema & de Lange, 2016). These findings are, therefore, exactly in line with effect-based account, where relevant paths are first perceptually organized, and the exact same representations can then form as basis for action control. We are looking forward to future studies that show such interactions in human participants.

### Imagery (and control) of complex actions

Imagery, especially for training purposes, often involves complex actions, such as the playing through of a martial arts kata, the complex motions of a platform dive, or even the different stages of a reach-to-grasp action (e.g., Driskell et al., 1994; Morris et al., 2005; Simonsmeier et al., 2018; Toth et al., 2020). In contrast, most empirical research on ideomotor control focusses on much simpler actions, which involve mostly discrete stimuli and responses, and which occur for the most part ballistically (e.g., button presses). It may, therefore, seem challenging for effect-based approaches to provide an account for how more complex actions are controlled—and, therefore, imagined.

We believe that, fundamentally, the difference between the simple actions used in classical ideomotor experiments and those more complex actions is probably smaller than one may assume, as even the simple button presses involve a carefully orchestrated sequence and press and release of multiple muscles, and even very simple action effects (such as visual cue) need to be distinguished in often complex ways (in terms, of shape, timing, location, etc.) from other unrelated stimuli that can occur at the same time. Thus, many of the difficulties that characterize complex actions are already present for the simple actions that have been subject of most research in the lab.

A central assumption is that the representations used for effect-based control of both simple and complex actions are organized in a hierarchical fashion, with higher level (distal) action goals at the top, and the required sequence of more proximal, body-related effects at the bottom, each robustly linked to the efferent/motor activities that would bring these effects about (e.g., Hommel et al., 2001, Hommel, 2009; for a recent review, see Moeller & Pfister, 2022). During execution, imaginistic control can then access—and modify—each of these more proximal action components to fulfill task requirements, such as making a less forceful button press than usual (Cao et al., 2020), varying the musical notes one is playing in a longer piece (e.g., Keller & Koch, 2008), or forming a more pronounced circular trajectory than usual during gesturing (e.g., Bach et al., 2010a). Moreover, during learning more complex action hierarchies can be built form the ground up from simpler elements and assembled into larger chunks (e.g., Moeller & Pfister, 2019). Indeed, a large variety of work now reveals effect-based control in more complex actions (e.g., throwing, Land, 2018), music sequences (e.g., Keller & Koch, 2008), gesturing (Bach et al., 2010a), motor sequence learning (e.g., Brown et al., 2022; Stöcker & Hoffmann, 2004) or in tool use (e.g., Massen & Prinz, 2009; Müsseler et al., 2008; for review and theoretical argument, see Badets & Osiurak, 2017), demonstrating that the described ideomotor principles do indeed apply such more complex behaviors.

When imagining such complex actions, imagery can then make use of the existing hierarchies, just as would be the case for imagery of more simple action. Similar to how agents would plan the execution of such actions, during imagery they can simply step through the sequence on the desired level of the hierarchy, either in terms of the major action steps (e.g., the route when driving home from work), or by accessing the more fundamental proximal representation that reflect the body movements required to achieve these goals, explaining perhaps the variety in how the timing of imagined actions matches or does not match the timing of overt execution (for a review, see Guillot & Collet, 2005). Indeed, studies that probe the cognitive representation of more complex skills show that the same hierarchies underlie imagery and overt action, so that less effective cognitive representations translate into less effective action (e.g., Schack & Mechsner, 2006; for a review, see Land et al., 2013), and that changes during imagery of a known motor skills induce analogous changes during overt action, and vice versa (Frank et al., 2014).

A related challenge is how imagery can model non-ballistic behaviors in which one’s required behavior in one step depends on what has happened in the previous steps, for example when correcting errors in performance, or when compensating for unexpected contributions from external influences (e.g., a partner’s movements in dance, or a wobble of the balance board one stands on). To flexibly control—and imagine—such dynamic responses to feedback, it has been argued that proximal effect representations may not only code discrete perceptual end states, but the *transitions* between a given (or imagined) perceptual state and an intended goal state (e.g., Kunde et al, 2017), or reduce the difference between both (i.e., prediction error minimization, Adams et al., 2013; Wolpert, 1997). Note, however, that feedback from one’s own actions—or from such external sources—is missing during imagery. Executive resources might therefore be necessary to supply such information (Glover & Baran, 2017; Martel & Glover, 2022), or imaginers might need to engage the “forward mode” of ideomotor learning to retrieve which outcomes the particular motor behaviors they imagined will have on the next components of their actions. Imagery of such multi-step actions, where one component depends on the previous ones, may therefore be a key example where motor imagery engages not only planning-related resources, but execution-related mechanisms to derive the specific outcomes one’s (imagined) motor behaviors will have.

### Evolution of motor imagery

The ability to imagine one’s actions undoubtedly brings substantial advantages to human behavior, allowing actors to play through different action alternatives in their mind before committing to one (e.g., Bennett, 2021), or to practice their skills offline before testing them “in the wild” (e.g., Simonsmeier et al., 2018; Toth et al., 2020). An interesting question is, therefore, when these imagery abilities, and the effect-based control of action on which they build, have evolved. Some theorists propose a co-evolution of ideomotor-like mechanisms together with abilities for action prediction, tool use and language (e.g., Badets & Osiurak, 2017). Others, in contrast, argue for an earlier emergence of effect-based control, and that even the behavior of simple organisms is well-described not in terms of a simple stimulus–response learning, but as an attempt to resolve, through the action, the mismatch between perceptually defined (homeostatic) goal states, and the organism’s actual state (i.e., active inference, Adams et al, 2013; Friston, 2013).

Several pieces of evidence point, in our mind, towards such an earlier evolution. For example, the phenomenon of outcome devaluation (Adams & Dickinson, 1981) marks the finding that even strongly overlearned stimulus–response associations (i.e., habits) do not lead to action when the associated outcomes are undesirable in the given situation. This and related phenomena are present in several animals, certainly in rodents (for a review, de Wit & Dickinson, 2009). At least for these animals therefore action planning already occurs on the basis of desired action outcomes and suggests an emergence of effect-based control already in non-human mammals.

Other findings suggest that also the ability to imagine behaviors and their outcomes, without overt motor output, is present in animals. It is known since Tolman (1948) that rats sometimes stop at critical junctions in a maze, as if considering—playing through in their mind—which path leads to the better outcome. Indeed, a plethora of studies since then suggest that is exactly what happens (for a review, see Reddish, 2016). Rats that exhibit this “vicarious trial and error” behavior are more successful in their navigation and arrive at the reward shortly after. Moreover, recording of hippocampal place cells confirm that during these instances the rat mentally travels along the paths within the maze, with associated firing of reward-related brain regions once the reward is (virtually) reached. This suggests again that rats, and potentially several species of birds (Sulikowski & Burke, 2015), have some abilities for motor imagery in the absence of action.

We are very much agnostic about this debate. For the account of motor imagery presented here, it is only necessary that these abilities are fully formed in humans. We note, however, that the ideas of effect-controlled action provide an excellent framework to account for the findings in animals above. We therefore suspect that what sets the remarkable human action planning abilities apart from that of animals is not the ability for effect-based control and imagery itself, which may be more fundamental than usually assumed, but the ability to *withhold* action when imagining. The key evolutionary step would then be the development of the prefrontal cortex, and the associated cognitive resources for response inhibition or up-regulating one’s motor threshold. These abilities would enable humans to imagine their actions in peace, without engaging in overt action, while still being able to derive the consequences these actions will have, therefore, opening up capacities for foresight, planning for (currently) counterfactual realities, and practicing skills in the absence of overt action.

## Conclusions

We have described how motor imagery can be conceptualized in effect-based views of action control. These views hold that actions are planned in term of their intended perceptual effects—how a successful action should look, feel, and sound—, which are then made reality by automatic motor processes. In such views, motor imagery reflects this imaginistic planning process, which is decoupled from execution. Imagery experience simply reflects the recall of one’s perceptual (visual, auditory, proprioceptive) experience with one’s actions, without requiring efferent (motoric) contributions. If these views are taken seriously, the term “motor imagery” becomes a misnomer; “effect imagery” might be a better description of how people imagine—and plan—their (motor) actions. The evidence reviewed here makes an explicit case for such an account.

## Footnotes

1 Many of the muscles that control finger movements are located in the arms, not the fingers. The particular sound when snapping the fingers is produced when the finger hits the palm of one’s hand, not by the fingers rubbing together. One makes a bicycle go left or right by leaning in this direction, not by moving the handlebars.

2 Our terminology throughout the manuscript follows the classic distinction between action planning and control, which is central to both effect-based (e.g., Hommel et al., 2001; Thomaschke et al. 2012; Wühr & Müsseler, 2001) and conventional accounts of motor control (e.g., Glover, 2004; Woodworth, 1899). While both planning and execution are important components of successful action, they subserve different functions. Planning captures primarily cognitive and volitional contributions, that is, the setting of action goals and relevant parameters (e.g., the speed of the action and the intended kinematics, trajectory). Control, in contrast, reflects the downstream (efferent) processes, which ensure that these goals are achieved, by guiding motor output towards the specified goal states. Of course, in practice, the boundary between both components is fluid, and research often observes a gradual transition between planning-related contributions at the start of the action, towards more control-related contributions at the end (for a review, see Glover, 2004). Moreover, recent proposals argue that even those downstream execution-related processes might be mediated by ideomotor-like mechanisms that move our effectors towards proprioceptively defined goal states (e.g., Adams et al., 2013)

## References

[CR1] Adams, C. D., & Dickinson, A. (1981). Instrumental responding following reinforcer devaluation. *The Quarterly Journal of Experimental Psychology,**33*, 109–121. 10.1080/14640748108400816 10.1080/14640748108400816

[CR2] Adams, R. A., Shipp, S., & Friston, K. J. (2013). Predictions not commands: Active inference in the motor system. *Brain Structure and Function,**218*(3), 611–643. 23129312 10.1007/s00429-012-0475-5 PMC3637647

[CR3] Anderson, M. (2010). Neural reuse: A fundamental organizational principle of the brain. *Behavioral and Brain Sciences,**33*(4), 245. 20964882 10.1017/S0140525X10000853

[CR4] Bach, P., Allami Khalaf, B., Tucker, M., & Ellis, R. (2014a). Planning-related motor processes underlie mental practice and imitation learning. *Journal of Experimental Psychology: General,**143*(3), 1277–1294. 24548280 10.1037/a0035604

[CR5] Bach, P., Griffiths, D., Weigelt, M., & Tipper, S. P. (2010a). Gesturing meaning. Non-action words activate the motor system. *Frontiers in Human Neuroscience,**4*, 214. 21120138 10.3389/fnhum.2010.00214 PMC2991204

[CR6] Bach, P., Knoblich, G., Gunter, T. C., Friederici, A. D., & Prinz, W. (2005). Action comprehension: Deriving spatial and functional relations. *Journal of Experimental Psychology: Human Perception & Performance,**31*(3), 465–479. 15982126 10.1037/0096-1523.31.3.465

[CR7] Bach, P., Nicholson, T., & Hudson, M. (2014b). The affordance-matching hypothesis: how objects guide action understanding and prediction. *Frontiers in Human Neuroscience*, 8 10.3389/fnhum.2014.00254 PMC4026748 24860468

[CR8] Bach, P., Peatfield, N. A., & Tipper, S. P. (2007). Focusing on body sites: The role of spatial attention in action perception. *Experimental Brain Research,**178*, 509–517. 17091293 10.1007/s00221-006-0756-4 PMC2079350

[CR9] Bach, P., Peelen, V. M., & Tipper, S. P. (2010b). On the role of object information in action observation: An fMRI study. *Cerebral Cortex,**20*(12), 2798–2809. 20231266 10.1093/cercor/bhq026 PMC2978238

[CR10] Badets, A., Koch, I., & Philipp, A. M. (2016). A review of ideomotor approaches to perception, cognition, action, and language: Advancing a cultural recycling hypothesis. *Psychological Research Psychologische Forschung,**80*(1), 1–15. 25535019 10.1007/s00426-014-0643-8

[CR11] Badets, A., & Osiurak, F. (2017). The ideomotor recycling theory for tool use, language, and foresight. *Experimental Brain Research,**235*(2), 365–377. 27815576 10.1007/s00221-016-4812-4

[CR12] Bennett, M. S. (2021). What behavioral abilities emerged at key milestones in human brain evolution? 13 hypotheses on the 600-million-year phylogenetic history of human intelligence. Frontiers in Psychology, 12 10.3389/fpsyg.2021.685853 PMC8358274 34393912

[CR13] Berthoz, A. (1996). The role of inhibition in the hierarchical gating of executed and imagined movements. *Cognitive Brain Research,**3*(2), 101–113. 8713551 10.1016/0926-6410(95)00035-6

[CR14] Bläsing, B., Tenenbaum, G., & Schack, T. (2009). The cognitive structure of movements in classical dance. *Psychology of Sport and Exercise,**10*, 350–360. 10.1016/j.psychsport.2008.10.001

[CR15] Brass, M., Bekkering, H., & Prinz, W. (2001). Movement observation affects movement execution in a simple response task. *Acta Psychologica,**106*(1–2), 3–22. 11256338 10.1016/S0001-6918(00)00024-X

[CR16] Brooks, R. W. (1995). Mental practice and the musician: A practical approach to practice. *Update: Applications of Research in Music Education,**13*(2), 4–8.

[CR17] Brown, R. M., Friedgen, E., & Koch, I. (2022). The role of action effects in motor sequence planning and execution: Exploring the influence of temporal and spatial effect anticipation. *Psychological Research Psychologische Forschung,**86*(4), 1078–1096. 34185146 10.1007/s00426-021-01525-2 PMC9090704

[CR18] Callow, N., Roberts, R., & Fawkes, J. Z. (2006). Effects of dynamic and static imagery on vividness of imagery skiing performance, and confidence. *Journal of Imagery Research in Sport and Physical Activity,**1*, 1–15. 10.2202/1932-0191.1001

[CR19] Cantergi, D., Awasthi, B., & Friedman, J. (2021). Moving objects by imagination? Amount of finger movement and pendulum length determine success in the Chevreul pendulum illusion. *Human Movement Science,**80*, 102879. 34607165 10.1016/j.humov.2021.102879

[CR20] Cao, L., Kunde, W., & Haendel, B. (2020). Rapid and accumulated modulation of action-effects on action. *Journal of Cognitive Neuroscience,**32*(12), 2333–2341. 32985944 10.1162/jocn_a_01633

[CR21] Carpenter, W.B. (1852). On the Influence of Suggestion in Modifying and directing Muscular Movement, independently of Volition. Royal Institution of Great Britain, (Proceedings), 147–153

[CR22] Chevreul, M. E. (1833). Lettre à M. Ampère sur une classe particulière de mouvements musculaires. *Review Des Deux Mondes,**2*, 258–266.

[CR23] Colton, J., Bach, P., Whalley, B., & Mitchell, C. (2018). Intention insertion: Activating an action’s perceptual consequences is sufficient to induce non-willed motor behaviour. *Journal of Experimental Psychology: General,**147*(8), 1256–1263. 29975079 10.1037/xge0000435

[CR24] Dahm, S. F., & Rieger, M. (2019). Is imagery better than reality? Performance in imagined dart throwing. *Human Movement Science,**66*, 38–52. 30913415 10.1016/j.humov.2019.03.005 PMC6520223

[CR25] de Lange, F. P., Helmich, R. C., & Toni, I. (2006). Posture influences motor imagery: An fMRI study. *NeuroImage,**33*(2), 609–617. 16959501 10.1016/j.neuroimage.2006.07.017

[CR26] de Lange, F. P., Rahnev, D. A., Donner, T. H., & Lau, H. (2013). Prestimulus oscillatory activity over motor cortex reflects perceptual expectations. *Journal of Neuroscience,**33*(4), 1400–1410. 23345216 10.1523/JNEUROSCI.1094-12.2013 PMC6618755

[CR27] de Wit, S., & Dickinson, A. (2009). Associative theories of goal-directed behaviour: A case for animal–human translational models. *Psychological Research PRPF,**73*(4), 463–476. 10.1007/s00426-009-0230-6 PMC2694930 19350272

[CR28] Decety, J., & Jeannerod, M. (1995). Mentally simulated movements in virtual reality: Does Fitt’s law hold in motor imagery? *Behavioural Brain Research,**72*(1–2), 127–134. 8788865 10.1016/0166-4328(96)00141-6

[CR29] Decety, J., Jeannerod, M., & Prablanc, C. (1989). The timing of mentally represented actions. *Behavioural Brain Research,**34*(1–2), 35–42. 2765170 10.1016/S0166-4328(89)80088-9

[CR30] Desmurget, M., & Grafton, S. (2000). Forward modeling allows feedback control for fast reaching movements. *Trends in Cognitive Sciences,**4*(11), 423–431. 11058820 10.1016/S1364-6613(00)01537-0

[CR31] di Pellegrino, G., Fadiga, L., Fogassi, L., Gallese, V., & Rizzolatti, G. (1992). Understanding motor events: A neurophysiological study. *Experimental Brain Research,**91*(1), 176–180. 1301372 10.1007/BF00230027

[CR32] Di Rienzo, F., Guillot, A., Daligault, S., Delpuech, C., Rode, G., & Collet, C. (2014). Motor inhibition during motor imagery: A MEG study with a quadriplegic patient. *Neurocase,**20*(5), 524–539. 23998364 10.1080/13554794.2013.826685

[CR33] Driskell, J., Copper, C., & Moran, A. (1994). Does mental practice enhance performance? *Journal of Applied Psychology,**79*, 481–492. 10.1037/0021-9010.79.4.481

[CR34] Drost, U. C., Rieger, M., Brass, M., Gunter, T. C., & Prinz, W. (2005a). Action–effect coupling in pianists. *Psychological Research Psychologische Forschung,**69*, 233–241. 15069559 10.1007/s00426-004-0175-8

[CR35] Drost, U. C., Rieger, M., Brass, M., Gunter, T. C., & Prinz, W. (2005b). When hearing turns into playing: Movement induction by auditory stimuli in pianists. *Quarterly Journal of Experimental Psychology,**58A*, 1376–1389. 10.1080/02724980443000610 16365945

[CR36] Easton, R. D., & Shor, R. E. (1975). Information processing analysis of the Chevreul pendulum illusion. *Journal of Experimental Psychology: Human Perception and Performance,**1*(3), 231–236. 10.1037/0096-1523.1.3.231 1202145 10.1037/0096-1523.1.3.231

[CR37] Easton, R. D., & Shor, R. E. (1976). An experimental analysis of the Chevreul pendulum Illusion. *The Journal of General Psychology,**95*(1), 111–125. 10.1080/00221309.1976.9710871 956790 10.1080/00221309.1976.9710871

[CR38] Easton, R. D., & Shor, R. E. (1977). Augmented and delayed feedback in the Chevreul pendulum illusion. *The Journal of General Psychology,**97*(2), 167–177. 10.1080/00221309.1977.9920835 28136225 10.1080/00221309.1977.9920835

[CR39] Effenberg, A. O., Fehse, U., Schmitz, G., Krueger, B., & Mechling, H. (2016). Movement sonification: Effects on motor learning beyond rhythmic adjustments. *Frontiers in Neuroscience,**10*(149), 67. 27303255 10.3389/fnins.2016.00219 PMC4883456

[CR40] Elsner, B., & Hommel, B. (2001). Effect anticipation and action control. *Journal of Experimental Psychology: Human Perception and Performance,**27*(1), 229. 11248937 10.1037//0096-1523.27.1.229

[CR41] Ferrari, P. F., Rozzi, S., & Fogassi, L. (2005). Mirror neurons responding to observation of actions made with tools in monkey ventral premotor cortex. *Journal of Cognitive Neuroscience,**17*(2), 212–226. 15811234 10.1162/0898929053124910

[CR42] Frank, C. (2014). Mental representation and learning in complex action: A perceptual-cognitive view on mental and physical practice (PhD thesis, Dr. rer. nat.). Universität Bielefeld

[CR43] Frank, C. (2016). Learning a motor action from within: Insights into the development of one’s action representation with mental and physical practice. In M. Raab, P. Wylleman, R. Seiler, A.-M. Elbe, & A. Hatzigeorgiadis (Eds.), *Sport and exercise psychology research from theory to practice* (pp. 91–121). Elsevier.

[CR44] Frank, C., Land, W. M., Popp, C., & Schack, T. (2014). Mental representation and mental practice: Experimental investigation on the functional links between motor memory and motor imagery. *PLoS One,**9*, e95175. 24743576 10.1371/journal.pone.0095175 PMC3990621

[CR45] Frank, C., Land, W. M., & Schack, T. (2013). Mental representation and learning: The influence of practice on the development of mental representation structure in complex action. *Psychology of Sport and Exercise,**14*, 353–361. 10.1016/j.psychsport.2012.12.001

[CR46] Friston, K. (2013). Life as we know it. *Journal of the Royal Society Interface,**10*(86), 20130475. 23825119 10.1098/rsif.2013.0475 PMC3730701

[CR47] Gallese, V., Fadiga, L., Fogassi, L., & Rizzolatti, G. (1996). Action recognition in the premotor cortex. *Brain,**119*(2), 593–609. 8800951 10.1093/brain/119.2.593

[CR48] Gandolla, M., et al. (2014). Re-thinking the role of motor cortex: Context-sensitive motor outputs? *NeuroImage,**91*, 366–374. 24440530 10.1016/j.neuroimage.2014.01.011 PMC3988837

[CR49] Glover, S., & Baran, M. (2017). The motor-cognitive model of motor imagery: Evidence from timing errors in simulated reaching and grasping. *Journal of Experimental Psychology: Human Perception and Performance,**43*(7), 1359. 28368162 10.1037/xhp0000389

[CR50] Guillot, A., & Collet, C. (2005). Duration of mentally simulated movement: A review. *Journal of Motor Behavior,**37*(1), 10–20. 15642689 10.3200/JMBR.37.1.10-20

[CR51] Guillot, A., Di Rienzo, F., MacIntyre, T., Moran, A., & Collet, C. (2012). Imagining is not doing but involves specific motor commands: A review of experimental data related to motor inhibition. *Frontiers in Human Neuroscience,**6*, 247. 22973214 10.3389/fnhum.2012.00247 PMC3433680

[CR52] Guillot, A., Lebon, F., & Collet, C. (2010). Electromyographic activity during motor imagery. In A. Guillot & C. Collet (Eds.), *The neurophysiological foundations of mental and motor imagery* (pp. 83–94). Oxford University Press.

[CR53] Guillot, A., Lebon, F., Rouffet, D., Champely, S., Doyon, J., & Collet, C. (2007). Muscular responses during motor imagery as a function of muscle contraction types. *International Journal of Psychophysiology,**66*(1), 18–27. 17590469 10.1016/j.ijpsycho.2007.05.009

[CR54] Guillot, A., Moschberger, K., & Collet, C. (2013). Coupling movement with imagery as a new perspective for motor imagery practice. *Behavioral and Brain Functions,**9*, 8. 23425312 10.1186/1744-9081-9-8 PMC3599464

[CR55] Hanakawa, T., Dimyan, M. A., & Hallett, M. (2008). Motor planning, imagery, and execution in the distributed motor network: A time-course study with functional MRI. *Cerebral Cortex,**18*(12), 2775–2788. 18359777 10.1093/cercor/bhn036 PMC2583155

[CR56] Harleß, E. (1861). Der Apparat des Willens. *Zeitschrift Für Philosophie Und Philosophische Kritik,**38*(2), 50–73.

[CR57] Hecht, H., Vogt, S., & Prinz, W. (2001). Motor learning enhances perceptual judgment: A case for action-perception transfer. *Psychological Research Psychologische Forschung,**65*(1), 3–14. 11505611 10.1007/s004260000043

[CR58] Hétu, S., Grégoire, M., Saimpont, A., Coll, M. P., Eugène, F., Michon, P. E., & Jackson, P. L. (2013). The neural network of motor imagery: An ALE meta-analysis. *Neuroscience & Biobehavioral Reviews,**37*(5), 930–949. 23583615 10.1016/j.neubiorev.2013.03.017

[CR59] Heuer, H. (1985). Wie wirkt mentale Übung? *Psychologische Rundschau,**36*(4), 191–200.

[CR60] Heuer, H., & Rapp, K. (2012). Adaptation to novel visuo-motor transformations: Further evidence of functional haptic neglect. *Experimental Brain Research,**218*(1), 129–140. 22328066 10.1007/s00221-012-3013-z

[CR61] Heuer, H., & Sülzenbrück, S. (2013). Towards mastery of complex visuo-motor transformations. *Frontiers in Human Neuroscience,**7*, 32. 23408336 10.3389/fnhum.2013.00032 PMC3570809

[CR62] Hommel, B. (2009). Action control according to TEC (theory of event coding). *Psychological Research PRPF,**73*(4), 512–526. 10.1007/s00426-009-0234-2 PMC2694931 19337749

[CR63] Hommel, B., Müsseler, J., Aschersleben, G., & Prinz, W. (2001). The theory of event coding (TEC): A framework for perception and action planning. *Behavioral and Brain Sciences,**24*(5), 849–878. 12239891 10.1017/S0140525X01000103

[CR64] Jacobson, E. (1931). Electrical measurements of neuromuscular states during mental activities. *American Journal of Physiology,**96*, 115–121. 10.1152/ajplegacy.1931.96.1.115

[CR65] Jacobson, E. (1932). Electrophysiology of mental activities. *American Journal of Psychology,**44*, 677–694. 10.2307/1414531

[CR66] James, W. (1890). *The principles of psychology* (Vol. 1). Henry Holt and Co.

[CR67] Janczyk, M., & Kunde, W. (2020). Dual tasking from a goal perspective. *Psychological Review,**127*(6), 1079–1096. 32538637 10.1037/rev0000222

[CR68] Janczyk, M., Pfister, R., Crognale, M. A., & Kunde, W. (2012). Effective rotations: Action effects determine the interplay of mental and manual rotations. *Journal of Experimental Psychology. General,**141*(3), 489–501. 22268853 10.1037/a0026997

[CR69] Jeannerod, M. (1994). The representing brain: Neural correlates of motor intention and imagery. *The Behavioral and Brain Sciences,**17*, 187. 10.1017/S0140525X00034026

[CR70] Jeannerod, M. (2001). Neural simulation of action: A unifying mechanism for motor cognition. *NeuroImage,**14*(1), S103–S109. 11373140 10.1006/nimg.2001.0832

[CR71] Jeannerod, M., & Decety, J. (1995). Mental motor imagery: A window into the representational stages of action. *Current Opinion in Neurobiology,**5*, 727–732. 8805419 10.1016/0959-4388(95)80099-9

[CR72] Kachergis, G., Wyatte, D., O’Reilly, R. C., de Kleijn, R., & Hommel, B. (2014). A continuous-time neural model for sequential action. *Philosophical Transactions of the Royal Society B: Biological Sciences,**369*(1655), 20130623. 10.1098/rstb.2013.0623 PMC4186241 25267830

[CR73] Karklinsky, M., & Flash, T. (2015). Timing of continuous motor imagery: The two-thirds power law originates in trajectory planning. *Journal of Neurophysiology,**113*(7), 2490–2499. 25609105 10.1152/jn.00421.2014 PMC4416550

[CR74] Kawato, M. (1999). Internal models for motor control and trajectory planning. *Current Opinion in Neurobiology,**9*(6), 718–727. 10607637 10.1016/S0959-4388(99)00028-8

[CR75] Keller, P. E., & Koch, I. (2008). Action planning in sequential skills: Relations to music performance. *Quarterly Journal of Experimental Psychology,**61*(2), 275–291. 10.1080/17470210601160864 17853237

[CR76] Kelso, J. A. S. (1995). *Dynamic patterns: The self-organization of brain and behavior*. MIT Press.

[CR77] Kilteni, K., Andersson, B. J., Houborg, C., & Ehrsson, H. H. (2018). Motor imagery involves predicting the sensory consequences of the imagined movement. *Nature Communications,**9*(1), 1–9. 10.1038/s41467-018-03989-0 PMC5915435 29691389

[CR78] Kohler, E., Keysers, C., Umilta, M. A., Fogassi, L., Gallese, V., & Rizzolatti, G. (2002). Hearing sounds, understanding actions: Action representation in mirror neurons. *Science,**297*(5582), 846–848. 12161656 10.1126/science.1070311

[CR79] Kühn, S., Keizer, A. W., Rombouts, S. A., & Hommel, B. (2011). The functional and neural mechanism of action preparation: Roles of EBA and FFA in voluntary action control. *Journal of Cognitive Neuroscience,**23*(1), 214–220. 20044885 10.1162/jocn.2010.21418

[CR80] Kunde, W. (2004). Response priming by supraliminal and subliminal action effects. *Psychological Research Psychologische Forschung,**68*(2), 91–96. 14634809 10.1007/s00426-003-0147-4

[CR81] Kunde, W., Koch, I., & Hoffmann, J. (2004). Anticipated action effects affect the selection, initiation, and execution of actions. *The Quarterly Journal of Experimental Psychology Section A,**57*(1), 87–106. 10.1080/02724980343000143 14681005

[CR82] Kunde, W., Müsseler, J., & Heuer, H. (2007). Spatial compatibility effects with tool use. *Human Factors,**49*(4), 661–670. 17702217 10.1518/001872007X215737

[CR83] Kunde, W., Schmidts, C., Wirth, R., & Herbort, O. (2017). Action effects are coded as transitions from current to future stimulation: Evidence from compatibility effects in tracking. *Journal of Experimental Psychology: Human Perception and Performance,**43*(3), 477. 27893272 10.1037/xhp0000311

[CR84] Lajoie, Y., Paillard, J., Teasdale, N., Bard, C., Fleury, M., Forget, R., et al. (1992). Mirror drawing in a deafferented patient and normal subjects: Visuoproprioceptive conflict. *Neurology,**42*, 1104–1106. 1579235 10.1212/WNL.42.5.1104

[CR85] Land, W. M. (2018). Priming of complex action via movement contingent sensory effects. *Human Movement Science,**61*, 135–143. 30092395 10.1016/j.humov.2018.08.001

[CR86] Land, W. M., Frank, C., & Schack, T. (2014). The impact of attentional focus on the development of skill representation in a complex action. *Psychology of Sport and Exercise,**15*, 30–38. 10.1016/j.psychsport.2013.09.006

[CR87] Land, W. M., Volchenkov, D., Bläsing, B. E., & Schack, T. (2013). From action representation to action execution: Exploring the links between cognitive and biomechanical levels of motor control. *Frontiers in Computational Neuroscience,**7*, 127. 24065915 10.3389/fncom.2013.00127 PMC3776155

[CR88] Liesner, M., & Kunde, W. (2020). Suppression of mutually incompatible proprioceptive and visual action effects in tool use. *PLoS ONE,**15*(11), e0242327. 33206706 10.1371/journal.pone.0242327 PMC7673520

[CR89] Linser, K., & Goschke, T. (2007). Unconscious modulation of the conscious experience of voluntary control. *Cognition,**104*(3), 459–475. 16996491 10.1016/j.cognition.2006.07.009

[CR90] Lotze, H. R. (1852). *Medicinische Psychologie oder Physiologie der Seele [Medical psychology or the physiology of the mind]*. Weidmann’sche Buchhandlung.

[CR91] Lotze, M., & Halsband, U. (2006). Motor Imagery. *Journal of Physiology - Paris,**99*, 386–395. 16716573 10.1016/j.jphysparis.2006.03.012

[CR92] Lutz, R. S. (2003). Covert muscle excitation is outflow from the central generation of motor imagery. *Behavioral Brain Research,**140*, 149–163. 10.1016/S0166-4328(02)00313-3 12644288

[CR93] Martel, M., & Glover, S. (2023). TMS over dorsolateral prefrontal cortex affects the timing of motor imagery but not overt action: Further support for the motor-cognitive model. *Behavioural Brain Research, 437*, 114125. 36167217 10.1016/j.bbr.2022.114125

[CR94] Maslovat, D., Chua, R., & Hodges, N. J. (2013). When unintended movements “leak” out: A startling acoustic stimulus can elicit a prepared response during motor imagery and action observation. *Neuropsychologia,**51*(5), 838–844. 23376312 10.1016/j.neuropsychologia.2013.01.016

[CR95] Massen, C., & Prinz, W. (2009). Movements, actions and tool-use actions: An ideomotor approach to imitation. *Philosophical Transactions of the Royal Society B: Biological Sciences,**364*(1528), 2349–2358. 10.1098/rstb.2009.0059 PMC2865071 19620106

[CR96] Mayer, J., & Hermann, H.-D. (2019). Kognitives Training im Sport. In J. Schüler, M. Wegner, & H. Plessner (Eds.), *Sportpsychologie* (pp. 463–478). Springer.

[CR97] Mechsner, F. (2003). Gestalt factors in human movement cordination. Gestalt Theory

[CR98] Mechsner, F., Kerzel, D., Knoblich, G., & Prinz, W. (2001). Perceptual basis of bimanual coordination. *Nature,**414*, 69–73. 11689944 10.1038/35102060

[CR99] Miall, R. C., & Wolpert, D. M. (1996). Forward models for physiological motor control. *Neural Networks,**9*(8), 1265–1279. 12662535 10.1016/S0893-6080(96)00035-4

[CR100] Moeller, B., & Frings, C. (2019). From simple to complex actions: Response–response bindings as a new approach to action sequences. *Journal of Experimental Psychology: General,**148*(1), 174. 30211579 10.1037/xge0000483

[CR101] Moeller, B., & Pfister, R. (2022). Ideomotor learning: Time to generalize a longstanding principle. *Neuroscience & Biobehavioral Reviews, 140*, 104782 35878792 10.1016/j.neubiorev.2022.104782

[CR102] Monaco, S., Malfatti, G., Culham, J. C., Cattaneo, L., & Turella, L. (2020). Decoding motor imagery and action planning in the early visual cortex: Overlapping but distinct neural mechanisms. *NeuroImage,**218*, 116981. 32454207 10.1016/j.neuroimage.2020.116981

[CR103] Moro, S. I., Tolboom, M., Khayat, P. S., & Roelfsema, P. R. (2010). Neuronal activity in the visual cortex reveals the temporal order of cognitive operations. *Journal of Neuroscience,**30*(48), 16293–16303. 21123575 10.1523/JNEUROSCI.1256-10.2010 PMC6634849

[CR104] Morris, T., Spittle, M., & Watt, A. (2005). *Imagery in sport*. Champaign: Human Kinetics.

[CR105] Munzert, J., & Krüger, B. (2018). Task-specificity of muscular responses during motor imagery: Peripheral physiological effects and the legacy of Edmund Jacobson. *Frontiers in Psychology,**9*, 1869. 30356730 10.3389/fpsyg.2018.01869 PMC6189391

[CR106] Munzert, J., Lorey, B., & Zentgraf, K. (2009). Cognitive motor processes: The role of motor imagery in the study of motor representations. *Brain Research Reviews,**60*(2), 306–326. 19167426 10.1016/j.brainresrev.2008.12.024

[CR107] Müsseler, J., & Hommel, B. (1997). Blindness to response-compatible stimuli. *Journal of Experimental Psychology: Human Perception and Performance,**23*(3), 861. 9180047 10.1037//0096-1523.23.3.861

[CR108] Müsseler, J., Kunde, W., Gausepohl, D., & Heuer, H. (2008). Does a tool eliminate spatial compatibility effects? *European Journal of Cognitive Psychology,**20*(2), 211–231. 10.1080/09541440701275815

[CR109] Naito, E. (2004). Sensing limb movements in the motor cortex: How humans sense limb movement. *The Neuroscientist,**10*(1), 73–82. 14987450 10.1177/1073858403259628

[CR110] Niziolek, C. A., Nagarajan, S. S., & Houde, J. F. (2013). What does motor efference copy represent? Evidence from speech production. *Journal of Neuroscience,**33*(41), 16110–16116. 24107944 10.1523/JNEUROSCI.2137-13.2013 PMC3792453

[CR111] Oosterhof, N. N., Tipper, S. P., & Downing, P. E. (2012). Visuo-motor imagery of specific manual actions: a multi-variate pattern analysis fMRI study. *NeuroImage,**63*(1), 262–271. 22766163 10.1016/j.neuroimage.2012.06.045

[CR112] O’Shea, H., & Moran, A. (2017). Does motor simulation theory explain the cognitive mechanisms underlying motor imagery? A critical review. *Frontiers in Human Neuroscience,**11*, 72. 28261079 10.3389/fnhum.2017.00072 PMC5313484

[CR01] Osiurak, F., & Badets, A. (2016). Tool use and affordance: Manipulation-based versus reasoning-based approaches. *Psychological Review, 123*(5), 534. 26881695 10.1037/rev0000027

[CR113] Papaxanthis, C., Paizis, C., White, O., Pozzo, T., & Stucchi, N. (2012). The relation between geometry and time in mental actions. *PLoS One,**7*(11), e51191. 23226487 10.1371/journal.pone.0051191 PMC3511381

[CR114] Paravlik, A., Slimani, M., Tod, D., Marusic, U., Milanovic, Z., & Pisot, R. (2018). Effects and dose-response relationships of motor imagery practice on strength development in healthy adult populations: A systematic review and meta-analysis. *Sports Medicine,**48*, 1165–1187. 29541965 10.1007/s40279-018-0874-8

[CR115] Pfister, R. (2019). Effect-based action control with body-related effects: Implications for empirical approaches to ideomotor action control. *Psychological Review,**126*(1), 153. 30604990 10.1037/rev0000140

[CR116] Pfister, R., Janczyk, M., Wirth, R., Dignath, D., & Kunde, W. (2014). Thinking with portals: Revisiting kinematic cues to intention. *Cogniton,**133*(2), 464–473. 10.1016/j.cognition.2014.07.012 25156629

[CR117] Prinz, W. (1992). Why don’t we perceive our brain states? *European Journal of Cognitive Psychology,**4*(1), 1–20. 10.1080/09541449208406240

[CR118] Prinz, W. (1997). Perception and action planning. *European Journal of Cognitive Psychology,**9*(2), 129–154. 10.1080/713752551

[CR119] Raffin, E., Mattout, J., Reilly, K. T., & Giraux, P. (2012). Disentangling motor execution from motor imagery with the phantom limb. *Brain,**135*(2), 582–595. 22345089 10.1093/brain/awr337

[CR120] Redish, A. D. (2016). Vicarious trial and error. *Nature Reviews Neuroscience,**17*(3), 147–159. 26891625 10.1038/nrn.2015.30 PMC5029271

[CR121] Reed, C. L. (2002). Chronometric comparisons of imagery to action: Visualizing versus physically performing springboard dives. *Memory & Cognition,**30*(8), 1169–1178. 12661849 10.3758/BF03213400

[CR122] Reichenbach, A., Franklin, D. W., Zatka-Haas, P., & Diedrichsen, J. (2014). A dedicated binding mechanism for the visual control of movement. *Current Biology,**24*(7), 780–785. 24631246 10.1016/j.cub.2014.02.030 PMC3988841

[CR124] Reiser, M., Büsch, D., & Munzert, J. (2011a). Strength gains by imagination of muscle actions with different ratios of physical to mental training. *Frontiers in Psychology, 2*, 194. 21897826 10.3389/fpsyg.2011.00194 PMC3158386

[CR126] Rieger, M., Martinez, F., & Wenke, D. (2011b). Imagery of errors in typing. *Cognition,**121*(2), 163–175. 21821234 10.1016/j.cognition.2011.07.005

[CR125] Rieger, M., Dahm, S. F., & Koch, I. (2017). Inhibition in motor imagery: A novel action mode switching paradigm. *Psychonomic Bulletin & Review,**24*(2), 459–466. 27363713 10.3758/s13423-016-1095-5 PMC5120687

[CR123] Rieger, M., Boe, S. G., Ingram, T., Bart, V.K.E., & Dahm, S. F. F. (under review). Action consequences in action imagery: internal prediction as an essential mechanism to detect errors. 10.1007/s00426-023-01812-0 PMC7616356 36961546

[CR127] Roelfsema, P. R., & de Lange, F. P. (2016). Early visual cortex as a multiscale cognitive blackboard. *Annual Review of Vision Science,**2*, 131–151. 28532363 10.1146/annurev-vision-111815-114443

[CR128] Roelfsema, P. R., Khayat, P. S., & Spekreijse, H. (2003). Subtask sequencing in the primary visual cortex. *Proceedings of the National Academy of Sciences,**100*(9), 5467–5472. 10.1073/pnas.0431051100 PMC154368 12695564

[CR129] Ryan, E. D., & Simons, J. (1981). Cognitive demand, imagery, and frequency of mental rehearsal as factors influencing acquisition of motor skills. *Journal of Sport Psychology,**3*, 35–45. 10.1123/jsp.3.1.35

[CR130] Ryan, E. D., & Simons, J. (1983). What is learned in mental practice of motor skills: A test of the cognitive-motor hypothesis. *Journal of Sport Psychology,**5*, 419–426. 10.1123/jsp.5.4.419

[CR131] Schack, T. (2004). The cognitive architecture of complex movement. *International Journal of Sport and Exercise Psychology,**2*, 403–438. 10.1080/1612197X.2004.9671753

[CR132] Schack, T. (2006). Mentales Training. In M. Tietjens & B. Strauss (Hrsg.), Handbuch Sportpsychologie (S. 254–261). Schorndorf: Hofmann

[CR133] Schack, T. (2020). Mental representation in action. A cognitive architecture approach. In G. Tenenbaum & R. Eklund (Eds.), *Handbook of Sport Psychology* (pp. 513–534). Hoboken: Wiley.

[CR134] Schack, T., Essig, K., Frank, C., & Koester, D. (2014). Mental representation and motor imagery training. *Frontiers in Human Neuroscience,**8*, 328. 24904368 10.3389/fnhum.2014.00328 PMC4033090

[CR135] Schack, T., & Frank, C. (2019). Ideomotor training. In D. Hackfort, R. Schinke, & B. Strauss (Eds.), *Dictionary of Sport Psychology: Sport, Exercise, and Performing Arts* (pp. 138–139). Elsevier.

[CR136] Schack, T., & Mechsner, F. (2006). Representation of motor skills in human long-term memory. *Neuroscience Letters,**391*(3), 77–81. 16266782 10.1016/j.neulet.2005.10.009

[CR137] Scheil, J., Kleinsorge, T., & Liefooghe, B. (2020). Motor imagery entails task-set inhibition. *Psychological Research Psychologische Forschung,**84*(6), 1729–1738. 30949789 10.1007/s00426-019-01183-5

[CR138] Schubotz, R. I. (2007). Prediction of external events with our motor system: Towards a new framework. *Trends in Cognitive Sciences,**11*(5), 211–218. 17383218 10.1016/j.tics.2007.02.006

[CR139] Shadmehr, R., Smith, M. A., & Krakauer, J. W. (2010). Error correction, sensory prediction, and adaptation in motor control. *Annual Review of Neuroscience,**33*, 89–108. 20367317 10.1146/annurev-neuro-060909-153135

[CR140] Shaw, W. A. (1938). The distribution of muscular action potentials during imaging. *Psychological Record,**2*, 195–216. 10.1007/BF03393216

[CR141] Shea, C. H., Buchanan, J. J., & Kennedy, D. M. (2016). Perception and action influences on discrete and reciprocal bimanual coordination. *Psychonomic Bulletin & Review,**23*(2), 361–386. 26282829 10.3758/s13423-015-0915-3

[CR142] Shenton, J. T., Schwoebel, J., & Coslett, H. B. (2004). Mental motor imagery and the body schema: Evidence for proprioceptive dominance. *Neuroscience Letters,**370*(1), 19–24. 15489010 10.1016/j.neulet.2004.07.053

[CR143] Shin, Y. K., Proctor, R. W., & Capaldi, E. J. (2010). A review of contemporary ideomotor theory. *Psychological Bulletin,**136*(6), 943. 20822210 10.1037/a0020541

[CR144] Simonsmeier, B. A., Andronie, M., Buecker, S., & Frank, C. (2021). The effects of imagery interventions in sports: A meta-analysis. *International Review of Sport and Exercise Psychology,**14*(1), 186–207. 10.1080/1750984X.2020.1780627

[CR145] Simonsmeier, B. A., Frank, C., Gubelmann, H., & Schneider, M. (2018). The effects of motor imagery training on performance and mental representation of 7- to 15-year old gymnasts of different levels of expertise. *Sport, Exercise, and Performance Psychology,**7*, 155–168. 10.1037/spy0000117

[CR146] Smith, D., Collins, D., & Holmes, P. (2003). Impact and mechanism of mental practice effects on strength. *International Journal of Sport and Exercise Psychology,**1*(3), 293–306. 10.1080/1612197X.2003.9671720

[CR147] Sperry, R. W. (1950). Neural basis of the spontaneous optokinetic response produced by visual inversion. *Journal of Comparative and Physiological Psychology,**43*(6), 482. 14794830 10.1037/h0055479

[CR148] Stinear, C. M., Byblow, W. D., Steyvers, M., Levin, O., & Swinnen, S. P. (2006). Kinesthetic, but not visual, motor imagery modulates corticomotor excitability. *Experimental Brain Research,**168*(1–2), 157–164. 16078024 10.1007/s00221-005-0078-y

[CR149] Stöcker, C., & Hoffmann, J. (2004). The ideomotor principle and motor sequence acquisition: Tone effects facilitate movement chunking. *Psychological Research Psychologische Forschung,**68*(2), 126–137. 14648225 10.1007/s00426-003-0150-9

[CR150] Sulikowski, D., & Burke, D. (2015). From the lab to the world: The paradigmatic assumption and the functional cognition of avian foraging. *Current Zoology,**61*(2), 328–340. 10.1093/czoolo/61.2.328

[CR151] Sun, D., Custers, R., Marien, H., Liefooghe, B., & Aarts, H. (2022). Examining mechanistic explanations for ideomotor effects. *Journal of Experimental Psychology: Human Perception and Performance, 48*(5), 458–466. 10.1037/xhp0000994 35377704

[CR152] Swinnen, S. P., Lee, T. D., Verschueren, S., Serrien, D. J., & Bogaerds, H. (1997). Interlimb coordination: Learning and transfer under different feedback conditions. *Human Movement Science,**16*(6), 749–785. 10.1016/S0167-9457(97)00020-1

[CR153] Tolman, E. C. (1948). Cognitive maps in rats and men. *Psychological Review,**55*(4), 189. 18870876 10.1037/h0061626

[CR154] Toovey, B. R. W., Seiss, E., & Sterr, A. (2021). Functional equivalence revisited: Costs and benefits of priming action with motor imagery and motor preparation. *Journal of Experimental Psychology: Human Perception and Performance,**47*(12), 1698. 34881954 10.1037/xhp0000966

[CR155] Toth, A. J., McNeill, E., Hayes, K., Moran, A. P., & Campbell, M. (2020). Does mental practice still enhance performance? A 24 Year follow-up and meta-analytic replication and extension. *Psychology of Sport and Exercise,**48*, 101672. 10.1016/j.psychsport.2020.101672

[CR156] Turvey, M. T. (1977). Preliminaries to a theory of action with reference to vision. In R. Shaw & J. Bransford (Eds.), *Perceiving, acting and knowing: Toward an ecological psychology* (pp. 211–265). Erlbaum.

[CR157] van Steenbergen, H., Warren, C. M., Kühn, S., de Wit, S., Wiers, R. W., & Hommel, B. (2017). Representational precision in visual cortex reveals outcome encoding and reward modulation during action preparation. *NeuroImage,**157*, 415–428. 28619654 10.1016/j.neuroimage.2017.06.012

[CR158] Wegner, D. M., Ansfield, M., & Pilloff, D. (1998). The putt and the pendulum: Ironic effects of the mental control of action. *Psychological Science,**9*(3), 196–199. 10.1111/1467-9280.00037

[CR159] Weinberg, R. (2008). Does imagery work? Effects on performance and mental skills. *Journal of Imagery Research in Sport and Physical Activity,**3*(1), 1–21. 10.2202/1932-0191.1025 10.2202/1932-0191.1025

[CR160] Wohlschläger, A. (1996). Mental rotation—a case of embodied action. In Embodied Cognition & Action. Papers from the 1996 Fall Symposium. Technical Report FS-96-05 (pp. 139–144)

[CR161] Wohlschläger, A. (2001). Mental object rotation and the planning of hand movements. *Perception & Psychophysics,**63*(4), 709–718. 11436739 10.3758/BF03194431

[CR162] Wohlschläger, A., & Wohlschläger, A. (1998). Mental and manual rotation. *Journal of Experimental Psychology: Human Perception and Performance,**24*(2), 397. 9606108 10.1037//0096-1523.24.2.397

[CR163] Wolpert, D. M. (1997). Computational approaches to motor control. *Trends in Cognitive Sciences,**1*(6), 209–216. 21223909 10.1016/S1364-6613(97)01070-X

[CR164] Woolfolk, R. L., Murphy, S. M., Gottesfeld, D., & Aitken, D. (1985a). Effects of mental rehearsal of task motor activity and mental depiction of task outcome on motor skill performance. *Journal of Sport Psychology,**7*, 191–197. 10.1123/jsp.7.2.191

[CR165] Woolfolk, R. L., Parrish, M. W., & Murphy, S. M. (1985b). The effects of positive and negative imagery on motor skill performance. *Cognitive Therapy and Research,**9*(3), 335–341. 10.1007/BF01183852

[CR166] Wulf, G. (2013). Attentional focus and motor learning: A review of 15 years. *International Review of Sport and Exercise Psychology,**6*(1), 77–104. 10.1080/1750984X.2012.723728

[CR167] Yue, G., & Cole, K. J. (1992). Strength increases from the motor program: Comparison of training with maximal voluntary and imagined muscle contractions. *Journal of Neurophysiology,**67*, 1114–1123. 1597701 10.1152/jn.1992.67.5.1114

[CR168] Zabicki, A., de Haas, B., Zentgraf, K., Stark, R., Munzert, J., & Krüger, B. (2017). Imagined and executed actions in the human motor system: Testing neural similarity between execution and imagery of actions with a multivariate approach. *Cerebral Cortex,**27*(9), 4523–4536. 27600847 10.1093/cercor/bhw257

[CR169] Ziessler, M., Nattkemper, D., & Vogt, S. (2012). The activation of effect codes in response preparation: New evidence from an indirect priming paradigm. *Frontiers in Psychology,**3*, 585. 23293623 10.3389/fpsyg.2012.00585 PMC3533231

[CR170] Zimmermann, M., Mars, R. B., De Lange, F. P., Toni, I., & Verhagen, L. (2018). Is the extrastriate body area part of the dorsal visuomotor stream? *Brain Structure and Function,**223*(1), 31–46. 28702735 10.1007/s00429-017-1469-0 PMC5772142

[CR171] Zimmermann, M., Verhagen, L., de Lange, F. P., & Toni, I. (2016). The extrastriate body area computes desired goal states during action planning. eNeuro, 3(2) 10.1523/ENEURO.0020-16.2016 PMC4821904 27066535

